# Zero standby power crop water-stress detector leading to the optimization of water usage and yield

**DOI:** 10.1038/s41598-022-16419-5

**Published:** 2022-07-23

**Authors:** Antea Risso, Vageeswar Rajaram, Sungho Kang, Sila Deniz Calisgan, Matilde Maria Pavese, Zhenyun Qian, Matteo Rinaldi

**Affiliations:** 1 Northeastern University, Boston, USA; 2 Northeastern SMART Center, Boston, USA

**Keywords:** Engineering, Materials science, Nanoscience and technology, Optics and photonics, Physics

## Abstract

Agricultural sensors are powerful tools to optimize crop productivity while conserving natural resources. Here we report a crop water-stress detector based on a plasmonically-enhanced micromechanical photoswitch capable of detecting water content in leaves that is lower than a predetermined threshold without consuming electrical power when the leaf is healthy. The detection mechanism exploits the energy in a specific narrow-spectral band of solar radiation reflected off leaves that is strongly correlated to the water content in plants. This biosensor relies on a spectrally selective infrared plasmonic absorber and a thermally sensitive micro-cantilever to harvest the reflected solar energy and further produce a digitized wakeup-bit only when the monitored leaf is water-stressed. In particular, we demonstrate that the detector activates a commercial water pump when a soybean plant is water-stressed. The 10-year battery lifetime of the proposed detector pave the way for the development of high-granularity, maintenance-free sensor networks for large-scale smart-farms.

## Introduction

The agriculture industry currently faces an urgent need to improve crop productivity in response to the rapidly increasing competition for arable land and exposure to climatic shocks. The population-driven need for food in the world is projected to increase by 70% by 2050, while land and natural resources such as water are becoming scarcer for farming^1^. Therefore, increasing the efficiency of food production is incredibly important to avoid global shortages. Famers make over 40 yield-impacting decisions each season, therefore there is a clear need to develop decision support tools that maximize economic returns^2^. The capability of acquiring comprehensive and near-real time data of plant health and environmental conditions with high granularity and translating them into actionable items would maximize the crop yield while conserving natural resources. Optimizing irrigation based on in-field spatiotemporal data reflecting crop water-need is one of the most important actions that can be taken in this regard. Existing state-of-the-art technologies for detecting plant water-stress, however, cannot be used to implement such a continuous monitoring of large-scale crop fields due to the prohibitive cost associated with maintenance and calibration^3^. Several agritech startups^4^ currently offer wireless sensor solutions to monitor the water content in the soil hoping to provide simple, actionable and accurate irrigation decisions. Nevertheless, they have been facing a fundamental challenge associated with remotely deployed sensor technologies: inadequate battery lifetime due to the high power consumed by the sensors in standby. Despite being marketed as low power solutions, most existing sensors still draw a standby current in the order of 1 mA with only a few exceptions being in the order of 10 s μA consumption^5,6^. The sensors typically stop working after few weeks in the field because of running out of battery power^3^. Some sensors employ a solar panel for energy harvesting to increase deployment time. However, this increases the overall sensor cost, complexity, and size due to the inclusion of the solar panel, its associated electronics and rechargeable batteries, which prevents the scaling of a sensor network towards high spatial resolution and large coverage.

Limitations also lie in the accuracy and ease of use of these sensors based on soil moisture measurement due to their indirect way of determining crop water-stress. The water contained in soil is only partially available to plants depending on the soil properties and the root system of the crop because i) the soil water retention capacity depends on the composition and compaction of the soil and ii) plants only draw water from the proximity of their root. Therefore, a soil moisture sensor must be pre-calibrated for the soil type and placed at the right depth close to the root (which varies per plant species and along time) in order to correctly estimate the available water to the plant^7^. Although the requirement for calibration varies between different types of soil moisture sensor, solutions that offer lite calibration process, low maintenance needs and high accuracy do not exist. For example, tensiometers require specialized calibration and frequent maintenance due to the strong dependance of soil moisture tension to the type of soil and the accumulation of air bubbles over time in the water tube. Granular matrix sensors require less maintenance, but their accuracy varies significantly between different soil types (e.g. less accurate in sandy soils). Similarly, time domain reflectometers feature easy calibration but are with low accuracy when the clay content and level of salinity are high in the soil. Their counterpart frequency domain reflectometers can be used in soils with high salinity, but it requires soil specific calibration^8,9^. Furthermore, the water content level in a plant depends on not only the soil moisture, but also the transpiration process and the capability of plant roots to extract water from the soil. The latter two are further affected by weather and the growth stage of the plant beyond just the species, which makes the translation from soil moisture to plant water-stress complicated and inherently inaccurate over time.

Hyperspectral imaging through satellite or drone is a more direct method of measuring crop water-need. The multispectral images of the crop canopy can be used to extract the information on plant water content because of the characteristic absorption of infrared (IR) radiation by the water in the leaves. Aerial imaging has advantages in terms of surveying a large area in a relatively short amount of time and almost no interference with the farming management. However, both satellite and drone-based approaches are characterized by a severely limited temporal resolution (~ days to weeks) due to either the long revisit period of satellite or the dependance on technical knowledge for drones’ use and on the meteorological conditions. In addition, the satellite-based approach is also characterized with a low spatial resolution due to the far distance for imaging. Moreover, further data processing and interpretation by experts are required to make decisions on the most effective irrigation plan. It can take up to several hours to derive useful information from aerial images making this technique far from being an accurate and convenient tool to address plant water needs^3^.

**Figure 1:**
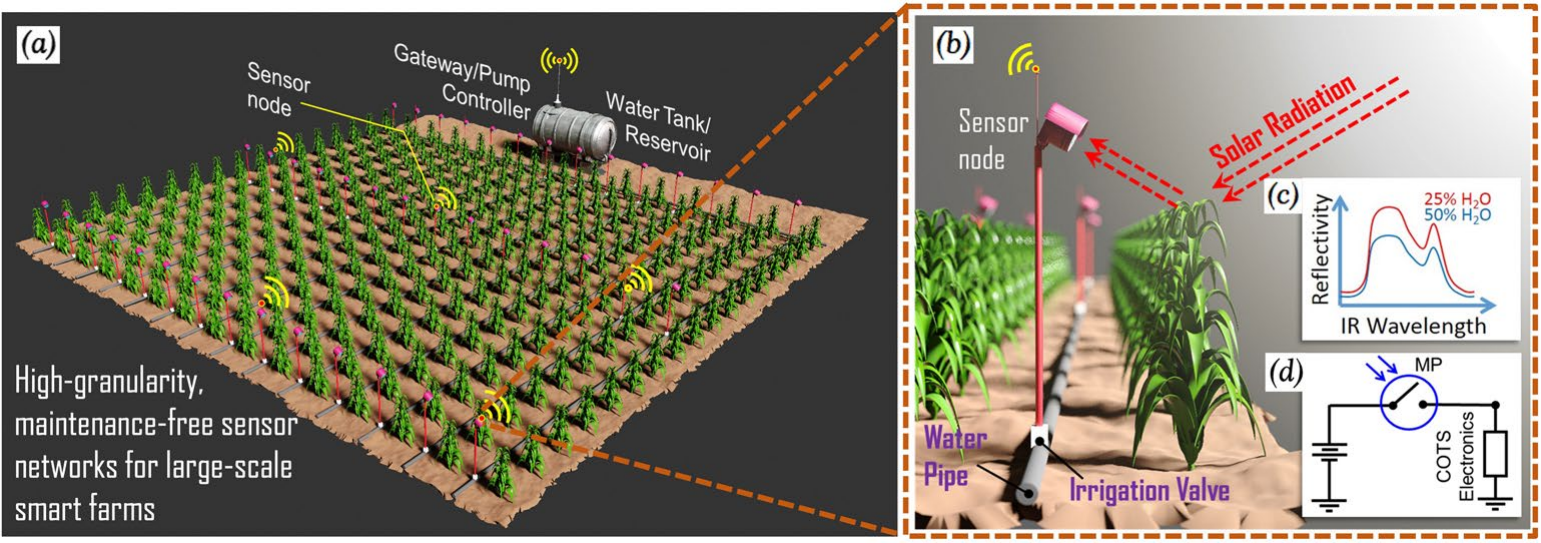
(**a**) Schematic illustration of our vision for a smart farm employing the proposed zero-power and low-cost sensor nodes in a crop field with high spatial granularity. (**b**) Schematic of the wireless sensor node used for non-contact water stress detection in plants. Upon plant water-stress detection, the switch closes and connects the battery to active electronics. e.g. a wireless transmitter that remotely triggers an automated irrigation system. The sensor remains otherwise OFF with zero drain on the battery. (**c**) Water stress-dependent IR reflectance characteristic of plants. As the plant becomes water stressed, the reflectance of the leaf in the SWIR wavelength region increases, causing a higher intensity of IR radiation to be reflected off the leaf surface when illuminated by the sun. (**d**) Simplified schematic of the circuit functionality showing the plasmonically-enhanced micromechanical photoswitch (PMP) controlling the current between the battery and the COTS electronics.

In this work, we propose to implement the optical-based direct measurement of plant water-stress at ground level with a network of spectrally-selective IR sensors (Fig. 1a,b). More specifically, we show that by leveraging an event-driven IR sensing capability, it is possible to realize the monitoring of water-stress with high accuracy as well as high spatial and temporal resolution while being long lasting and low cost, which simultaneously addresses all the challenges associated with the existing soil moisture sensing and aerial hyperspectral imaging methods. The concept of event-driven sensing was recently proposed to bridge the energy gap of powering large-scale unattended ground sensor networks by developing a new class of completely-passive micromechanical sensors capable of detecting and distinguishing the signal of interests without using active electronics^10,11^. The water-stress sensor technology presented here selectively harvest the IR radiation reflected off from leaves and then use it to generate a wake-up bit to activate a water pump when the water content in the leaves is below a pre-determined threshold. The detection mechanism relies on the dependence of the leaf reflectance on the plant water-stress caused by the strong IR absorption of water in the short-wavelength infrared (SWIR) region (1.3–1.6 μm) (Fig. 1c). When the plant is water-stressed, the reduced absorption by water makes the surface of leaves more reflective. Therefore, when illuminated by the sunlight, the reflected IR power from the leaves in the SWIR band can be exploited as a signature for detecting plant water-stress.

The zero-standby power plant water-stress detector is based on a plasmonically-enhanced micromechanical photoswitch (PMP)^11–19^ previously reported by our group. Differently from all previous work, a PMP operating in the SWIR band suitable for detecting and digitizing the IR light reflected off a plant leaf is demonstrated in this work. We show that only when the reflected IR exceeds a predetermined power threshold that corresponds to a low level of leaf relative water content (RWC), the PMP turns ON and activates the irrigation or wireless transmission, otherwise the entire system remains dormant with a power consumption below 1 nW, 5 orders of magnitude lower than the state of the art. The drastically reduced standby power consumption directly translate to a 10-year battery lifetime of a wireless water-stress sensor node (Fig. 1d), which leads to more than 4 times lower maintenance cost associated with battery replacement. Compared to soil moisture sensors, the technology also features high accuracy and being easy to use thanks to the direct measurement of water stress in plant and the actionable output. Therefore, we believe the technology is ideal for the development of high-granularity, maintenance-free sensor networks for auto-irrigation systems in large-scale smart farms.

## Results

### Device design and characterization

**Figure 2:**
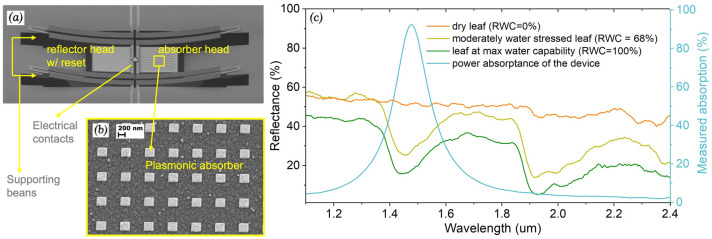
(**a**) Scanning electron microscope (SEM) image of the fabricated PMP. The right cantilever has a SWIR plasmonic absorber while the left one reflects IR and has an in-built microheater for reset. The contacts of the PMP are located in the middle. (**b**) Close-up of the absorber surface. The lateral dimensions of the metal patches determine absorption wavelength. (**c**) Fourier Transform-IR (FTIR) spectrometer measurement of the absorptance spectrum of the fabricated device and of the reflectance spectra of dry (RWC = 0%), visually moderately water stressed (RWC = 68%) and non-stressed (RWC = 100%) leaves. Absorption peak of the device matches well with the water absorption valley region of the leaf’s reflectance spectra.

A PMP is composed of a pair of microcantilevers facing each other supported by four pairs of thermally sensitive bimaterial folded beams (Fig. 2a,b). A narrowband absorber is integrated with one of the cantilevers while a reflector is integrated in the other, which creates a temperature difference between the two sides when the device is illuminated by an IR radiation matching the absorbing band. When exposed to above threshold IR intensity, the two electrical contacts between the cantilevers are brought into contact forming an electrical path due to the thermal expansion in the supporting beans. Thanks to the symmetrical design, the device is immune to ambient temperature changes. The working principle and fabrication process of the device are explained in more detail in the Supplementary Section 1 and^11^. Differently from previous work, the PMP presented here incorporates a high-efficiency narrowband plasmonic absorber (η ~ 93%, 150 nm bandwidth) targeting the “water absorption valley” (centered at 1.47 μm) of a leaf’s reflection IR spectrum, where there is a strong dependence of the reflectance on the RWC of a leaf (Fig. 2c)^20^. A resistive microheater is also added to the reflector head to realize a reset function in the event of the contacts are latched due to adhesion force after turning ON from a detection. Application of a short voltage pulse across the heater causes the reflector head to be thermally actuated downward and away from the absorber head, which reopens the contacts after a detection. The resistance of the heater was measured to be 53.7 kΩ by applying a 10 mV voltage across the heater and monitoring the current passing through it. Pull-in voltage was found to be 21.5 V. The OFF and ON state resistances were measured to be > 10 GΩ (instrument noise limited) and ~ 5 kΩ respectively^17^.

### Water-stress detection

In this section, we show that the sensor is able to activate a commercial off-the-shelf (COTS) water pump^21^ automatically when the plant being monitored becomes water stressed. Before performing the tests to demonstrate water-stress detection using the PMP, the leaf reflectance was first characterized as a function of its RWC as described in the Supplementary Section 2. Results are shown in Fig. 4a (red curve).

**Figure 3:**
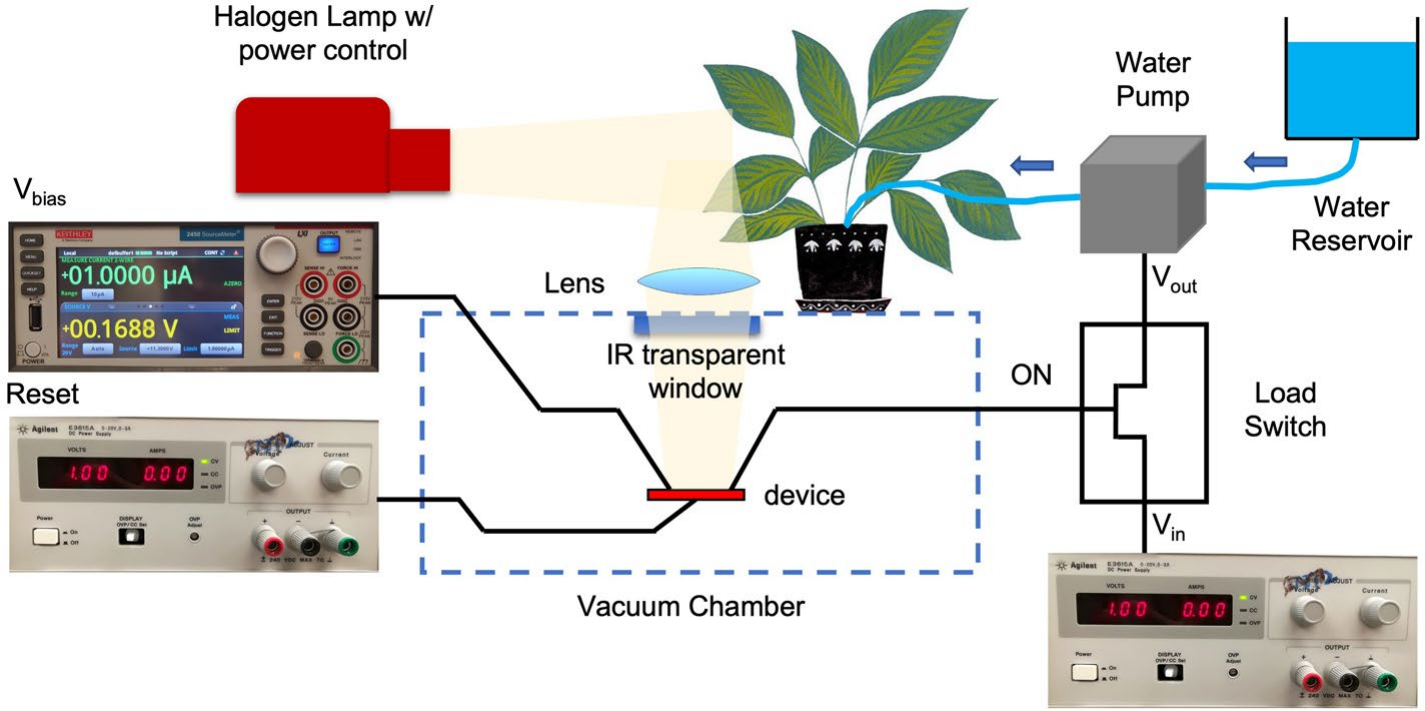
Schematic of the experimental setup used to demonstrate the zero standby power SWIR soybean plant water stress detection. It includes a Quartz tungsten halogen lamp (sunlight simulator), a soybean alive plant, a lens, a vacuum chamber where the PMP was placed, a load switch, a water reservoir and a water pump. The PMP was connected to a source-meter to apply the V_bias_ and to monitor the switch current. It was also connected to a separate DC supply to apply the reset pulse. Another DC supply was used as V_in_ of the load switch. A moderate water-stress condition of the plant (RWC = 68%) resulted in the PMP closing and, as a consequence, triggering the ON port of the load switch. At that point V_out_ = V_in_ resulting in the activation of the water pump which was programmed to pump 20 ml of water over 20 s.

Then, the experimental setup for demonstrating the proposed technology was assembled in a laboratory setting. Figure 3 shows the setup where a broadband IR illumination source (quartz tungsten halogen lamp) was used to simulate sunlight. The light was directed towards a leaf on a live soybean plant (*Glycine Max*) which was placed on top of a vacuum probe station containing the PMP. The leaf was secured on a holder tilted at 45° with respect to the source to reflect light to the PMP through an IR-transparent window (made of calcium fluoride—CaF_2_). A lens on top of the window (also CaF2) was used to focus the reflected light from the leaf on to the PMP. A source meter (Agilent 2450), connected to one end of the PMP through a DC probe, was used to apply a bias voltage of 19 V across the contacts while monitoring the switch current. The bias voltage acts as a passive method to scale down the PMP’s threshold to the required value^17^. The other end of the PMP was linked through a DC probe to the ON pin of an ultra-low leakage load switch (SiP32421). The load switch was used to handle the high current (~ 600 mA) drawn by the load (COTS pump) because the PMP by itself can handle only up to 200 µA^11^. The input voltage to the load switch V_in_ was connected to another DC supply set at 5.8 V (the pump’s operating voltage). The output voltage V_out_ was confirmed to be equal to 0 V when the PMP is in the open/standby state and equal to V_in_ when the PMP was closed, and the ON pin was high. The detailed electrical circuit is described in the Supplementary Section 3. When the switch was turned ON the source meter reading abruptly changed from ~ 4 nA (i.e. noise floor of the instrument) to ~ 10 µA (internally limited). A 1 V pulse (~ 100 ms) from a separate DC source applied to the reset heater (R = 53.7 kΩ) was used to reopen the contacts to guarantee repeatable testing.

The IR power reflected from a soybean leaf with varying RWC was first characterized by substituting the PMP in the experimental setup (Fig. 3) with a commercial IR sensor, from which the required PMP threshold was found to be ~ 209 nW corresponding to a moderate water-stressed state (RWC = 68%). This RWC was chosen as the moderate water-stressed level since the plant was still easily recoverable at this state if watered. The fabricated PMP was then placed in the chamber and fine-tuned to have a threshold of 209 nW by applying a bias voltage of 19 V^17^.

**Figure 4:**
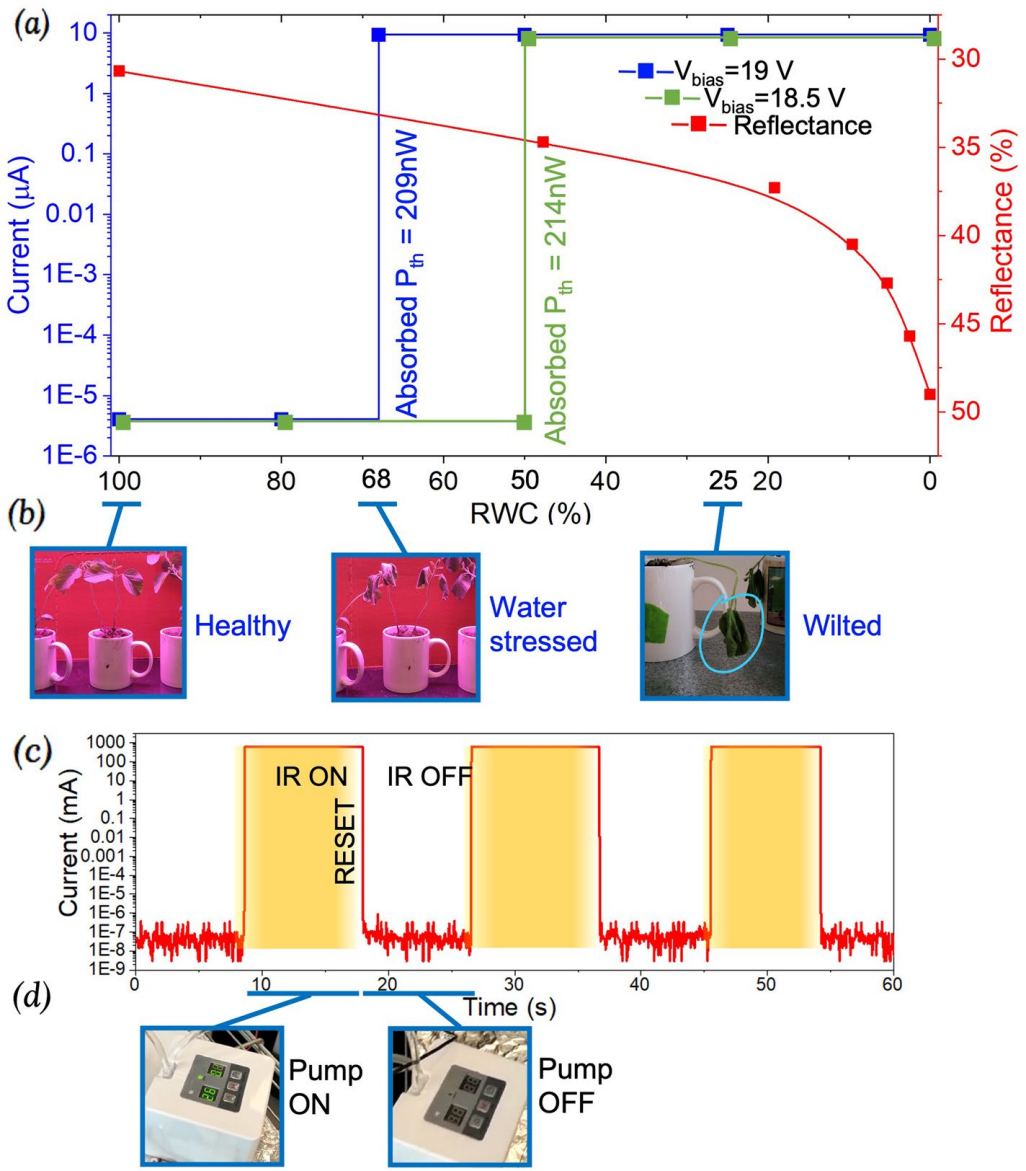
(**a**) Experimental result of the current flowing through the PMP with two different bias voltages and reflectance of the leaf as a function of the relative water content (RWC) of the leaf. (**b**) The tested soybean plant at different RWCs (100%, 68%, and 25%). (**c**) Measured current through the entire system in response to chopped IR radiation reflected off the leaf at RWC = 68%. The PMP latches ON after each detection and the reset function returns the switch to an OFF state until triggered ON again by above-threshold IR. (**d**) The water pump turns ON when the PMP closes as a response to IR radiation reflected off the leaf at RWC = 68%. After the reset, the water pump remains OFF until triggered ON again by an above-threshold IR radiation.

The PMP was then sequentially exposed to the soybean plant with RWC decreasing from 100 to 25% while the current in the device was monitored. As expected, the device reliably turned ON only when exposed to samples with RWC ≤ 68% as shown by the abrupt (> 6 order of magnitude) current change in Fig. 4a (blue curve). After detection of RWC = 68%, a piece of paper has been used as a shutter on top of the vacuum chamber’s window, in order to reset the device while allowing the leaf to dry to even lower values of RWC. Once reached the desired level of RWC, the shutter was removed and the device successfully detected lower levels of water stress in the leaf. The device remained completely off with zero standby leakage (instrument noise limited to ~ 10^–5^ µA) for higher RWCs as required. Using the source meter, an overall system current of ~ 600 mA was measured when the PMP was triggered ON and the pump was activated, and only ~ 120 pA when the PMP was off, and the system was at standby. It is notable that this represents a > 230,000 times improvement over the standalone pump (with no water-stress sensing function) which is normally operated on a duty-cycle and had a standby current of 28.4 µA. It is also worth noting that with a reflectance change of 3.2% in the range from 100% RWC to 68% RWC, the device is able to differentiate between the IR power levels at these two RWCs (189.3 nW versus 209 nW).

An additional experiment was performed and an RWC of 50% has been identified as the minimum RWC for which, if a plant is watered, it recovers (i.e. its water-stressed wilting point) (more details in Supplementary Section 4). To simulate the scenario corresponding to the limit of detection for our sensor, the PMP was fine-tuned at a bias voltage of 18.5 V in order to detect 214 nW corresponding to a leaf reflectance change of 4% and turning on at RWC = 50% (Fig. 4a (green curve),b).

This case has been shown purely for demonstrative purposes as it corresponds to the maximum reflectance change that can be targeted by the sensor for recoverability of the plant. However, it is important to highlight that in a real scenario if the stress level is set at the wilting point, it will likely affect yield and trigger chronic physiological damages^22^.

Nevertheless, the implementation of irrigation management techniques such as the optimized regulated deficit irrigation (ORDI) are beginning to be explored as the limited water reservoirs are getting more valuable due to climate change and increasing demand. ORDI distributes the total available water based on the needs at each growing stage. In this scenario, the demonstrated tunability of our sensor can be used to arbitrary choose an RWC level far from the wilting point but under low or moderate water stress to optimize water usage while avoiding chronic physiological damages^23^. The reasons for a deficit irrigation can be government incentives for water conservation, shortage in water supply^24^ and/or for the purpose of achieving a tastier product (mostly fruits) filled with vitamins and antioxidants^25^.

As a test for repeatability, Fig. 4c shows the measured current through the PMP in response to chopped IR radiation reflected off the water stressed leaf at RWC = 68%. Due to the pull-in voltage effect, the PMP latches ON after each detection. To reopen the switch for the next detection, a 1 V pulse (~ 100 ms) applied to the reset heater (53.7 kΩ) returns the switch to an open and OFF state until triggered ON again by above-threshold IR when the shutter is removed from the top of the vacuum chamber’s window. Each time the PMP is triggered ON the water pump turns on as shown in Fig. 4d.

Compared to a conventional commercial low-power battery-powered water stress sensor such as a soil moisture sensor (ECOWITT WN51^6^) which was measured to have a power consumption of 60 µW at standby, our water stress sensor features a 75,000 times reduced power consumption of only 720 pW in standby. While the soil moisture sensor is duty cycled, the PMP turns ON only when sensing water stress and therefore its frequency at which it turns on varies according to the growth stage and season (e.g. soybean plants need more water during pod development and early seed fill as well as during drier/warmer weather). In a hypothetical in-field situation, with a Panasonic CR2354 coin battery (capacity ~ 560 mAh), the lifetime of the water stress sensor based on a PMP was estimated and compared to the Ecowitt soil moisture sensor (when they are connected to a same wireless transmitter with the soil moisture sensor duty cycled every 20 min). The calculation shows that while the state-of-the-art soil moisture sensor would last less than one year (~ 8 months) the water stress sensor based on a PMP results in almost 4-years lifetime. Compared to the state-of-the-art soil moisture sensor, the proposed zero-power crop water-stress detector can continuously monitor the plant water stress while enabling 5.7 times longer battery lifetime and higher accuracy thanks to its direct measurement scheme. A comparison showing twice the improvement of the proposed sensor over soil moisture sensors in the case of activating a water pump is also included in the Supplementary Section 5.

### Potential impact on yield improvement and water saving

We believe that the unique combination of high accuracy and ultra-low power consumption of the proposed water stress sensor technology will enable a significant water use efficiency (WUE, defined as grain produced per unit of water used by the crop) improvement that cannot be achieved with soil moisture sensors in a large scale. Studies have shown that precise irrigation based on the growth stage and real-time water stress level in a plant (e.g., soybeans in the case of^26^) leads to a maximized yield. Due to the indirect measurement, soil moisture sensors are unable to guide the irrigation system to precisely control the RWC of a plant within an optimum range in different growth stages. In this context, the demonstrated IR-based direct RWC measurement and the threshold tuning capability of the proposed water stress sensors are ideal for monitoring a plant’s water need in real time and further generating actionable items (digitized output bits) to control the irrigation systems. The threshold of the sensors in this case can be adjusted along the growth of the plant to ensure a RWC value always maintained above the optimum level regardless of the change of soil property and temperature^27^, weather conditions and root depth that have to be taken into account when a soil moisture sensor is employed. On the other hand, it has been reported that maximized water saving can be achieved through a dripping irrigation system with high-granularity sensor input (~ 5 × 5 m^2^ area)^28^. Although soil moisture sensors are used in trial fields for such studies, there are no commercially available products can be deployed in such a density in a large scale due to the prohibitive cost associated with sensor hardware and maintenance. By replacing the soil moisture sensors with the proposed zero-power water stress sensors, it is expected that the same or better result on water saving can be achieved and with greatly reduced labor cost spent on calibration and battery replacement hence a promising solution for large scale deployment.

## Discussion

One key aspect of the presented device concept is to use sunlight as the IR source in order to keep the sensor node fully passive at standby. However, the sunlight is a constantly varying power source making it challenging to rely on thresholding method to gauge the water stress in the leaf. Therefore, in order to practically implement the presented sensing mechanism in the field, a stable and calibrated light source (in a greenhouse for example) or additional sunlight sensor is required. Unlike the case for IR sensing, detection and digitizing of light intensity in visible spectrum with ultra-low power consumption is less challenging with existing technologies. For instance, a miniature and self-sustaining sunlight digitizer circuit was demonstrated^29^, which can be used in conjunction with the sensor technology presented here to mitigate the problem related to sunlight variation. Other possible challenges associated with outdoor development are from the environmental conditions such as large temperature change, wind, rain, dust, fog, etc. In principle, the temperature compensation mechanism in PMP^30^ and the operating wavelength at SWIR spectral band help address some of the challenges in certain extent. For example, IR light can pass through smoke and fog with little attenuation thanks to their longer wavelength. Future work will focus on the design of a robust sensor system and deployment scheme based on the technology for field test.

In conclusion, we have demonstrated a first-of-its-kind zero-power water stress sensor based on the monitoring of leaf reflectance rise when its RWC drops. The use of sunlight as the IR source together with the sensor’s event-driven IR sensing capability make it fully passive at standby which leads to a drastically extended sensor lifetime compared to state-of-the-art low-power soil moisture sensors. The direct measurement scheme contributes to a high accuracy without the need of soil dependent calibration, which is critical for precision agriculture aiming at maximized yield with reduced water usage. These features directly translate to a low maintenance cost and a high return of investment making the proposed technology an ideal candidate for the implementation of high-granularity water stress sensor networks suitable for large-scale smart farms.

## Online methods

### Plant selection and growing conditions

We chose to perform our tests on Soybean (Glycine Max) due to its fast growth rate, quick response to water-stress and large amount of relevant data available in literature. More specifically, the selected seeds are labeled “Non-GMO, Gluten Free Laura Soybeans” and they are sold by Sanlinx Inc. The Lot Number is NJKS-19-LA and the Harvest Date was October 15, 2019. The soybean plant was grown in an enclosed Delux Smart Grow Closet in our lab, which was fitted with air filters (to avoid cross-contamination between the lab and closet), continuous airflow, a thermo/hygrometer and grow lights. The hygrometer, a thermometer and a Kind LED K3 Series 2 XL450 grow light allowed us to keep the plant under constant humidity (~ 30%) and temperature (~ 26 °C) as well as to enable perpetual growing cycles. A DIY Micro Automatic Drip Irrigation Kit was used to water the plants daily (300 mL/day). All methods were performed in accordance with relevant guidelines and regulations.

### RWC as a measure of water stress

Among the many indices used in literature to model reflectance spectra versus the water content of the leaf, we chose the Relative Water Content (RWC) for two main reasons. Firstly, it is relatively easy to obtain directly from the weight of the leaf (directly dependent on the leaf water content) and is easy to calculate. The second reason is because we found consistent RWC literature connecting the reflectivity of Glycine Max to its water content therefore allowing a solid comparison of our results with results of already existing techniques.

The measurements were done with circular cut leaf discs of 3 cm diameter (We avoided large veins when cutting). The formula used was: RWC (%) = [(W − DW)/(TW − DW)] × 100 where W = weight of the cut leaf disc, TW is the weight of the leaf disc after being immersed in water in a closed petri dish to reach full turgidity in 3-4 h under normal lab conditions (we measured TW = 0.126 g), DW is the mass of the leaf disc dried at 80C for 24 h and weighed (measured DW = 0.015 g).

Generally, the RWC was 98% for fully turgid transpiring leaves, 60% to 70% for the initial wilting stage and 30% to 40% for severely desiccated and dying leaves.

## Supplementary Information

Supplementary Information.

## Data Availability

The data that support the plots within this paper and other findings of this study are available from the corresponding authors upon reasonable request.
