# Supplementary material for: Zero standby power crop water-stress detector leading to the optimization of water usage and yield
Source: Sci Rep. 2022 Jul 23;12:12603. doi: 10.1038/s41598-022-16419-5 (PMC9308807; doi:10.1038/s41598-022-16419-5)
Supplement: Supplementary file 1 — Supplementary Information. [file 41598_2022_16419_MOESM1_ESM.docx]

Zero Standby Power Crop Water-Stress Detector Leading to the Optimization of Water Usage and Yield


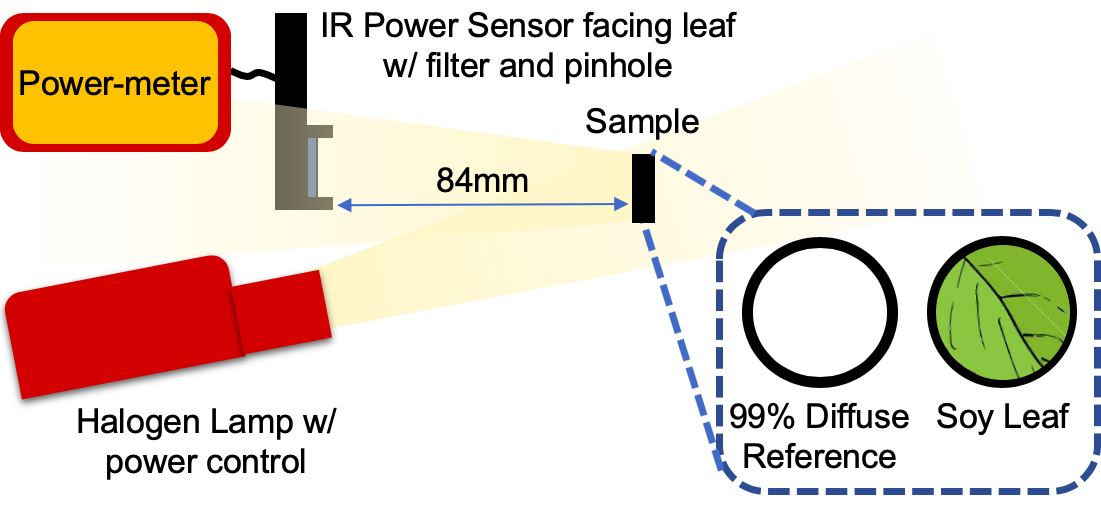

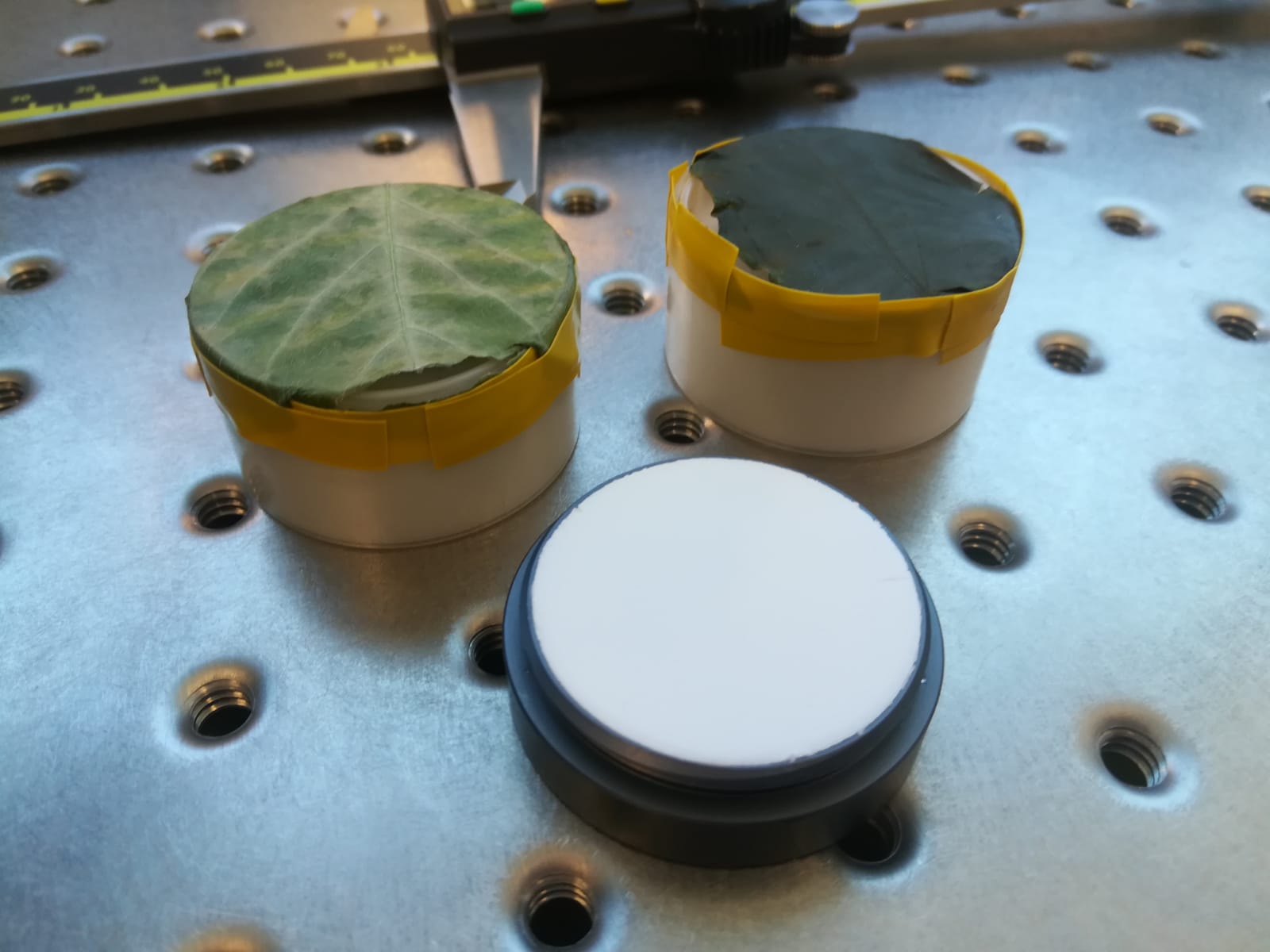


Figure S1. Experimental setup used to measure a soybean leaf reflectance versus its RWC. The setup includes a light source with diffuser, a Thorlabs S132C power sensor, an Ocean Optics diffuse reflectance standard and a sample holder positioned 84mm from the detector.


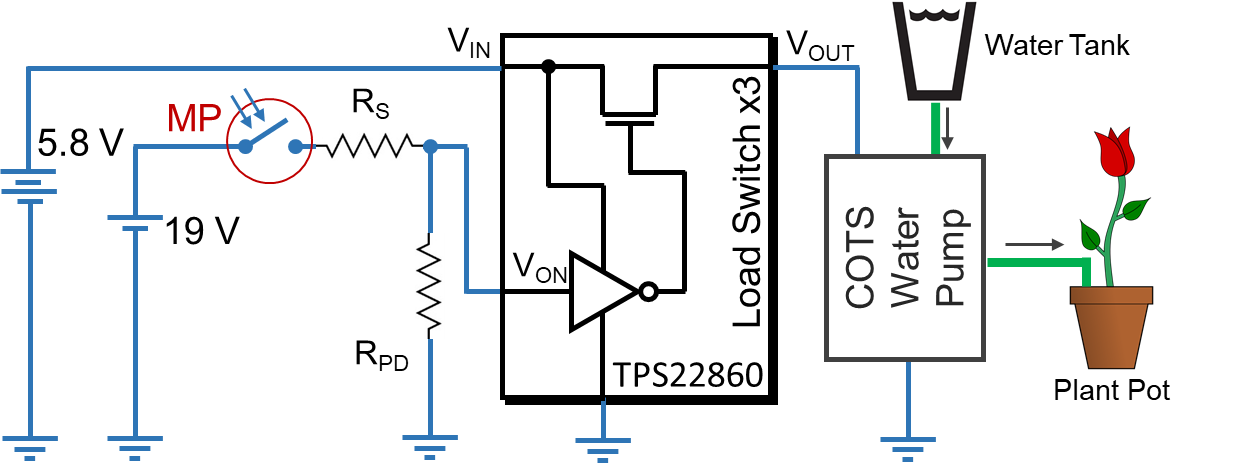


Figure S2. Schematic of the Load-switch-based sensor circuit used to demonstrate water stress detection


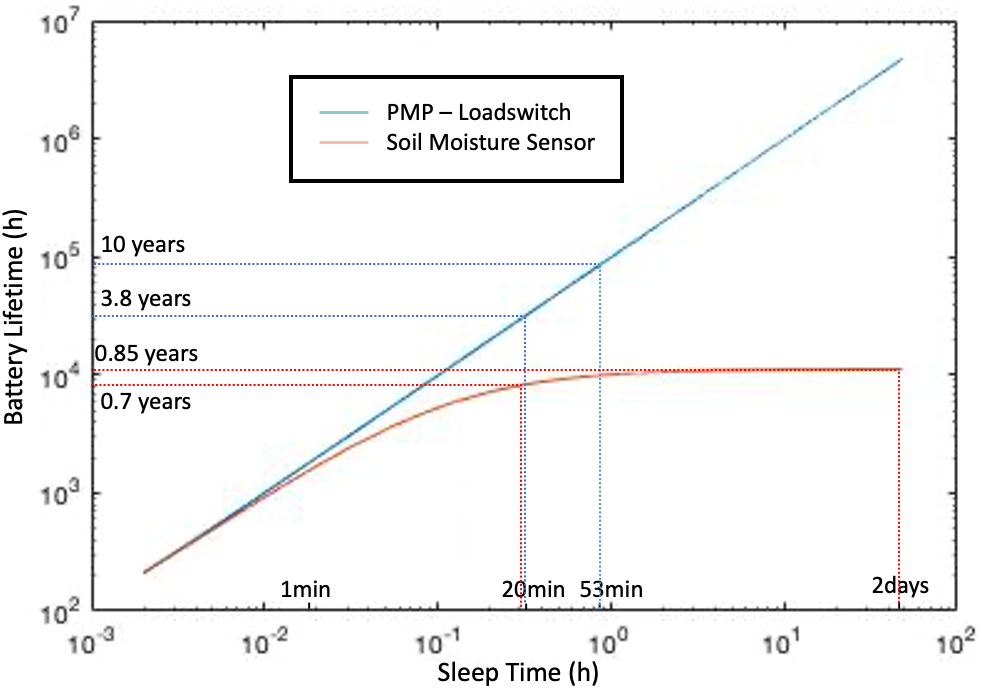

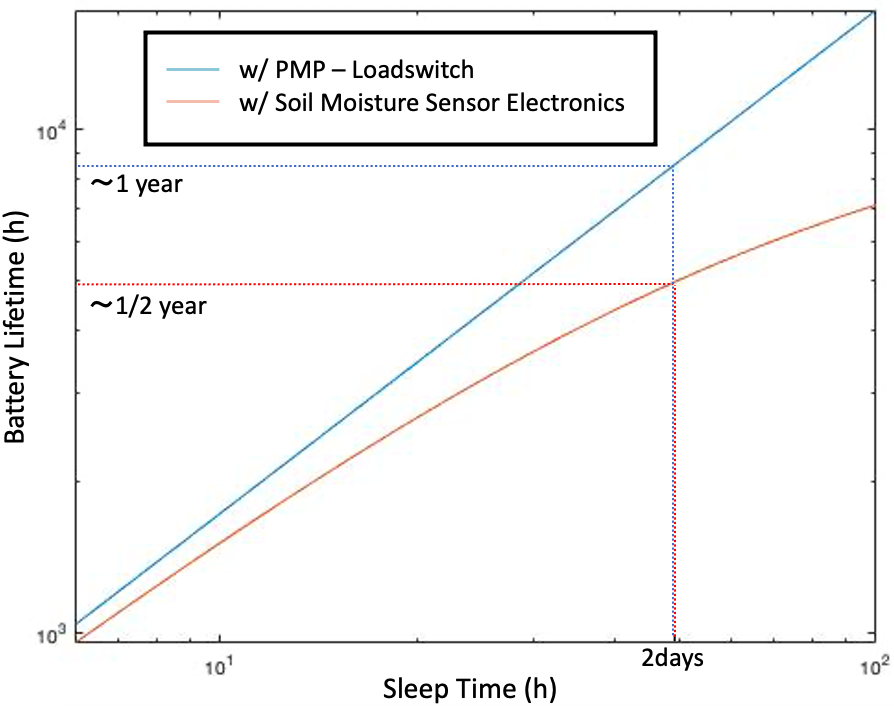


Figure S3. (a) Battery life versus sleep time of the state-of-the-art soil moisture sensor versus the proposed zero power crop water-stress detector, when maintaining the same ON time and wireless transmitter. (b) Battery life versus sleep time of the water pump attached to the PMP through the load switch and the hypothetical situation in which the water pump would be attached to the soil moisture sensor electronics.

**Section 1: Design, Fabrication and characterization of the PMP**

The PMP comprises a pair of symmetric suspended cantilevers, each composed of an IR absorbing (or reflecting) head and an inner and outer pair of thermally sensitive bi-material legs separated by a thermal isolation link. The IR absorbing head with the integrated plasmonic absorber selectively converts impinging optical power to heat. Upon the absorption of narrowband SWIR radiation, a large and fast increase of temperature in the corresponding inner pair of legs results in a downward displacement of the cantilever, bringing a high-stiffness platinum (Pt) tip into contact with another contact on the reflector head when the temperature rise is high enough (i.e., when the incident power is above its designed threshold). The working mechanism is described in further detail in^1^. It is worth noting that the switch remains open with a physical gap until it absorbs sufficient SWIR radiation (i.e. its threshold power) reflected from the leaf regardless of the change in ambient temperature, thanks to the symmetric design of the two cantilevers. The sub-micron air gap between the contacts at standby translates directly into zero leakage current and therefore zero-standby power consumption. A microheater is also integrated on the reflector head to act as a reset function.

The key features of the switch structure are as follows: (a) The plasmonic absorber on one cantilever head and a gold (Au) reflector on the other, (b) a bowl-shaped Pt contact, (c) bi-material beams and (c) an embedded microheater under the reflector head for reset function. The plasmonic absorber comprises a metal-insulator-metal stack made of a bottom reﬂector (5-50-5 nm thick Ti-Pt-Ti), a thin dielectric spacer (50 nm thick SiO2) and an array of Au patches (5-50-5 nm thick Ti-Au-Pt nanostructures) whose absorption wavelength can be tuned lithographically [1]. When exposed to IR, plasmonically-enhanced electromagnetic coupling causes electrical currents in the metal layer of the absorber which in turn causes heating from ohmic losses. This heat is conducted to the bi-material inner legs where it is confined to by the thermal isolation. The heat causes the downward bending of the inner legs due to differential thermal expansion between the Al and SiO_2_ layer and the subsequent vertical displacement of the absorber head puts the top contact tip (a bowl-shaped high-stiffness Pt structure) in contact with the metal pad on the opposite reflector head. The Pt tip and the contact pad are separated by a physical air gap that prevents any subthreshold currents unlike CMOS based switches. Any other unwanted deflection that can affect the contact gap and thus the threshold, for example the change in ambient temperature or residual stress, is compensated for by the symmetric folded design of the structure [2].

A 13-mask microfabrication process was used to fabricate the new reflectance-based water-stress sensors, which included one mask-less step of electron-beam lithography for the plasmonic absorber. The fabrication process flow is described in further detail in supplementary of^1^ with the difference here being the integration of a new reset functionality through a heater in the lower head as a first deposited layer, as well as a customized lithographic tuning of the absorber wavelength to detect water-stress in the leaf. A high resistivity 4-inch Si wafer was selected to enable the use of the bottom heater without current leakage through the substrate.

## **Section 2: Reflectance versus RWC**

Before performing the tests to demonstrate water-stress detection using the PMP, the leaf reflectance was first characterized as a function of its RWC. The experimental setup to characterize a soybean leaf reflectance for varying degrees of water stress is shown in Fig. S1. A circular piece of leaf was chopped from the plant and left overnight in deionized water. This way, the experiment started from the “wet boundary” (its maximum level of water content, where RWC=100%). Once the leaf was removed from the deionized water and pat-dried, it was taped on a circular plastic sample holder. The plastic holder and tape were weighed first without and then with the leaf attached, and for the rest of the experiment the leaf was left attached to the holder (the accurate weight of the leaf was needed to calculate the RWC at each step). The data was collected for the leaf first at the “wet boundary” (0th hour) and then re-measured each successive hour, for five hours. The water-stress was simulated by leaving the leaves in a dry cabinet at 38% humidity. To find their “dry boundary” (RWC = 0%), as a final step in the experiment, they were placed in an oven at ~80°C until completely dry and then re-measured. Overall, seven data points were collected for RWC and Reflectance. The experimental setup for the reflectance measurement contained the following:

A light source with a SWIR-transparent diffuser to evenly distribute the light on the leaf.

A Thorlabs S132C power sensor (700-1800 nm range of detection) set to detect at 1550 nm, with 1550 nm bandpass filter (80 nm bandwidth) and a pinhole (which delimits the area of the sensor and simulates an actual PMP) at its aperture. It was located perpendicular to the leaf (0° angle), just above the light source. It was connected to a Thorlabs PM100D Power console to record the readings.

An Ocean Optics diffuse reflectance standard (95% reflectance) as reference (the leaf is a diffuse surface).

A sample holder positioned 84 mm far away from the detector. The 95% reflectance reference and the leaf on the holder had approximately the same area (38.75mm diameter) and were both placed at the same distance (84mm) with respect to the detector when examined. Each time a power measurement was taken, everything was first removed from the holder and the power detector was zeroed. This way we guaranteed that the power detected from the surroundings was not taken into consideration during the collection of the data. First, the power reflected from the reference was measured knowing that it corresponded to 95% reflectivity. Next the reference was removed from the holder and replaced with the leaf and the power reflected by it was measured. The ratio of the power measured with the leaf to the power measured with the reflectance standard was considered as the reflectance.

As expected, based on literature, the chopped Soybean leaf dried up very fast: the RWC index dropped rapidly (from 100% to 47.7%) within only one hour of exposure to the dry environment in the Delux Smart Grow Closet. In fact, after the first hour the leaf looked already unhealthy (close to its wilting point). The measured change in reflectance after the first hour was 4%.

At 1550 nm, literature states that for a Soybean plant the reflectance goes from ~ 30% reflectance for the wet boundary to ~ 55% reflectance for the dry one. In our case the measured values go from ~ 30% to ~ 49%. We consider the results close enough to already existing values and the difference may be caused by the difference in the type/subspecies of Soybean plant.

Since we used a 1.55 um filter with bandwidth of 80 nm, that was available, we extrapolated the values for 1.47 μm with bandwidth 150nm (to match our device’s absorption) based on the FTIR measured absorption data.

**Section 3: Design rationale for the circuit**

A commercial DIY Micro Automatic Drip Irrigation Kit operating at 5.8 V was used to supply water to the soybean plant when water-stressed. Connecting it to the source meter as the power supply allowed us to measure the current while it was operating. In order to integrate the water pump in the system, three Texas instruments TPS22860 Ultra Low-Leakage Load Switches, each with a maximum current capacity of 200 mA, were stacked in parallel to handle the 600 mA current requirement of the pump (Fig. S2). The ON/OFF input of the load switch (V_ON_) was connected to the PMP through a simple voltage divider composed of a 1.5 M Ω resistance (R_S_) connected to the voltage coming from the PMP (19 V when closed, 0 V when open) and a 470k resistance connected to ground (R_PD_). The output of the voltage divider (6 V when closed, 0 V when open) was connected to the ON/OFF input of the load switch. The Vin pin of the lead switch was connected to a DC supply providing 5.8 V and a current limited to 600 mA not to damage the load switch. Finally, by connecting the V_OUT_ pin of the load switch to the water pump, whenever the PMP detected water-stress in the plant and closed, the V_ON_ input pin received 6 V changing Vout from 0 V to V_IN_=5.8 V eventually resulting in the activation of the pump and the plant being watered.

**Section 4: Characterization of Wilting Point and Maximum Reflectance Change for Recoverability**

An additional experiment was performed in order to identify the minimum RWC for which, if a plant is watered, it recovers (i.e. its water-stressed wilting point). Five soybean plants at a same growth stage with an initial state of 100% RWC, each in its own pot, were prepared for this experiment. With time, the RWC gradually lowered due to natural evapotranspiration and the first plant was watered at an RWC=63%, whereupon it was found to recover to its full RWC of 100%. By the time the second plant was watered its RWC was 57%. This plant also successfully recovered. This process was subsequently repeated for the next three plants (at RWC of 50%, 47% and 35%). It was found that the last two did not recover upon watering them and thus it was concluded that that the minimum RWC for which, if watered, the plant recovers, is 50%. This corresponds to a leaf reflectance change of 5% and is expected to be the upper limit of detection for our sensors. Thus, if the aim is to minimize water usage to the maximum extent, the PMP can be tuned to turn on at RWC=50%.

This case has been shown purely for demonstrative purposes as it corresponds to the maximum reflectance change that can be targeted by the sensor for recoverability of the plant. However, it is important to highlight that in a real scenario if the stress level is set at the wilting point, it will likely affect yield and trigger chronic physiological damages [3].

Nevertheless, the implementation of irrigation management techniques such as the optimized regulated deficit irrigation (ORDI) are beginning to be explored as the limited water reservoirs are getting more valuable due to climate change and increasing demand. ORDI distributes the total available water based on the needs at each growing stage. In this scenario, the demonstrated tunability of our sensor can be used to arbitrary choose an RWC level far from the wilting point but under low or moderate water stress to optimize water usage while avoiding chronic physiological damages [4].

**Section 5: Battery Life versus Sleep time. Comparison with a duty-cycled soil-moisture-sensor**

Fig. S3a show the PMP compared to the Ecowitt soil moisture sensor when connected to a coin Panasonic CR2354 coin battery (capacity 560mAh) and to the same wireless transmitter. While the commercially available soil moisture sensor currently has a duty cycle to activate every 72 seconds, the PMP turns ON only when sensing water stress and therefore it changes its sleep time according to the growth stage and season. Overall, while the state-of-the-art soil moisture sensor lasts ~one year, the PMP, thanks to its wake-up function, would activate less frequently than every 72 seconds since, after being watered, a plant takes hours to water-stress again (resulting in a 10 years PMP’s battery lifetime as long as the plant dries to the chosen RWC threshold level less often than every 50 minutes). In such scenario, the water stress sensing function of the PMP results in its lifetime only limited by the shelf life of the battery itself. Furthermore, to increase the sleep time of the duty cycle of the state-of-the-art soil moisture sensor, would result in a saturated 1-year lifetime while increasingly reducing accuracy. Fig. S3a shows a hypothetical in-field situation where, compared to the state-of-the-art soil moisture sensor, the proposed zero power crop water-stress detector, when maintaining the same ON time (with a 20-minutes sleep time) and the same wireless transmitter as the soil moisture sensor, enables 5.7 times longer battery lifetime while guarantying accuracy thanks to its water-stress sensing function. Fig. S3b shows the comparison of the water pump attached to the PMP through the load switch versus the hypothetical situation in which the water pump would be attached to the soil moisture sensor electronics. In the case where the water pump has the standby power of the PMP-load switch, if the water pump would be activated every other day, the battery life of the system would be of 1 year while, for the same sleep time, if the water pump would be attached to the soil moisture sensor electronics, it would be reduced to only half a year.

**References**

1. S. Kang, Z. Qian, V. Rajaram, S. D. Calisgan, A. Alu’, and M. Rinaldi, **“**[Ultra-narrowband Metamaterial Absorbers for High Spectral Resolution Infrared Spectroscopy](https://doi.org/10.1002/adom.201801236)**”**, *Advanced Optical Materials*, vol. 1801236, p. 1801236, Nov. 2018. doi:10.1002/adom.201801236
2. Z. Qian, S. Kang, V. Rajaram, C. Cassella, N. E. McGruer, and M. Rinaldi, “Zero-power infrared digitizers based on plasmonically enhanced micromechanical photoswitches,” Nat. Nanotechnol., vol. 12, no. 10, pp. 969–973, Sep. 2017.
3. J.J. Pardo, A. Sánchez-Virosta, B.C. Léllis, A. Domínguez, A. Martínez-Romero, “Physiological basis to assess barley response to optimized regulated deficit 2 irrigation for limited volumes of water (ORDIL)”, SSRN, Apr 2022.
4. A Sánchez-Virosta, B.C Léllis, J.J Pardo, A Martínez-Romero, D Sánchez-Gómez, A Domínguez, “Functional response of garlic to optimized regulated deficit irrigation (ORDI) across crop stages and years: Is physiological performance impaired at the most sensitive stages to water deficit?”, Agricultural Water Management, Volume 228, 105886, Feb 2020.
